# Traumatic Brain Injury and Neuropsychiatric Consequences

**DOI:** 10.1192/j.eurpsy.2025.1248

**Published:** 2025-08-26

**Authors:** B. Peixoto, M. Cruz, V. Ustares

**Affiliations:** 1 Psychiatry, Hospital do Divino Espirito Santo, Ponta Delgada, Portugal

## Abstract

**Introduction:**

Post-traumatic brain injury (TBI) is a leading cause of long-term neuropsychiatric consequences, which can significantly impair quality of life and complicate recovery. These consequences include cognitive deficits in memory, attention, and executive functions, depression, anxiety, apathy, obsessive-compulsive disorder, psychosis, personality changes, impulsivity and agitation in moderate to severe cases. In cases of repetitive head trauma, there is a higher risk of developing chronic traumatic encephalopathy, a neurodegenerative disorder charaterized by dementia and motor abnormalities. Understanding the spectrum and prevalence of these disturbances is crucial for developing effective management and rehabilitation strategies.

**Objectives:**

The aim of this study is to review the prevalence and treatment of neuropsychiatric consequences of traumatic brain injury.

**Methods:**

The authors did a non-systematic review of the current literature.

**Results:**

This review revealed that neuropsychiatric consequences are present in approximately 80% of post-TBI patients, depending on the severity of the injury and the time passed from the trauma. Depression and anxiety disorders were the most prevalent, followed by cognitive deficits. Personality changes, impulsivity, and agitation were commonly observed in moderate to severe cases. Factors such as the extent of frontal lobe injury and the time elapsed since the injury were strongly associated with the severity of neuropsychiatric symptoms. There is no pharmalogical treatment approved by the Food and Drug Administration (FDA), but the literature recommends the use of SSRI (in cases of depression), beta blokers (in high doses for agitation), antipsychotics (only if necessary and with caution, because these patients are more propense to the side effects and it can impair cognition and motor rehabilitation) and anticonvulsants (carbamazepine or valproate in cases of agitation or irritability).

**Conclusions:**

Neuropsychiatric complications of TBI are common and have significant implications for long-term recovery and quality of life. Early identification and comprehensive management strategies, including pharmacological and psychological interventions, are essential to address these challenges and improve patient outcomes. Further research is needed to refine diagnostic tools and therapeutic approaches tailored to the neuropsychiatric consequences of TBI.

**Disclosure of Interest:**

None Declared

